# Supplementary material for: Learning curve and surgical outcome of robotic assisted colorectal surgery with ERAS program
Source: Sci Rep. 2022 Nov 29;12:20566. doi: 10.1038/s41598-022-24665-w (PMC9709162; doi:10.1038/s41598-022-24665-w)
Supplement: Supplementary file 2 — Supplementary Information 2. [file 41598_2022_24665_MOESM2_ESM.zip › RAL Raw data-Ñ[▒K/090/HS_ñw╝╨░O▒Kñσ.pdf]

正式摘要(電子簽章：0000002188842042)	□【讀取】憑證	
W76 / W76 □英文標題(注意:無英文姓名-請補病人基本檔!)		

臺中榮民總醫院

出院病歷摘要

轉入號 : 轉出號 :

[redacted]

(8)流水編號	
(9)入院日期	110/06/04 (2021) 直腸外科 W76 - 09
(10)轉科日期	
(11)出院日期	110/06/09 (2021) 住院天數計 5 日

診斷

- 入院 :1. S-Colon Cancer, c T3 N0 M0, Stage IIA
  2. HTN
  2. angina ,r/o CAD
  3. Sjogren syndrome with 1) ANA 2) SSA 3) xerostomia proved by scialocintigaphy 4. hypergammaglobulinemia
  5. Cat allergy with allergic rhinitis
- 出院 :1. S-Colon Cancer, c T3 N0 M0, Stage IIA s/p robotic assisted anterior resection on 2021/06/05
  2. HTN
  3. angina ,r/o CAD
  4. Sjogren syndrome with 1) ANA 2) SSA 3) xerostomia proved by scialocintigaphy 4. hypergammaglobulinemia
  5. Cat allergy with allergic rhinitis

主訴

- RUQ dullness pain, especially after food since 2021/03.

病史

- This 52 y/o female with a history of

  1.HTN
  2.angina ,r/o CAD
  3.Sjogren syndrome with 1) ANA 2) SSA 3) xerostomia proved by scialocintigaphy 4) hypergammaglobulinemia
  4.Cat allergy with allergic rhinitis
  suffered from freqent RUQ dullness pain, especially after food since 2021/03. She went to GI OPD for help where colonscopy showed S-colon CA. As a result, she was refered to CRS OPD for help and she this time, she was amitted for operation.

體檢發現

-
  - General appearance :
    - easy looking , conscious clear , without cardiopulmonary distress
    - Vital signs自動帶入date 體溫(BT) 脈搏(P) 呼吸(R) 血壓(BP) 身高(BH) 體重(BW) BMI
      20210604 35.5℃ 71次/分 16次/分 102/66mmHg 164.3cm 62.2kg 23.0
    - Vital signs: B.P. 102/66 mmHg , P.R. 71 /min , R.R. 16 /min , B.T. 35.1 ℃
  - HEENT :
    - Eyes: no anemic conjunctiva, no icteric sclera, normal EOM
    - ENT: no otorrhea, no rhinorrhea, no erythematous mucosa, no injected tonsil, no deformity
  - Neck: supple, freely movable, trachea midline,no deformity, no engorged jugular vein , no palpable thyroid or lymph node enlargement
  - Chest:
    - Inspection: Grossly normal, symmetric and fully expansion, no accessory muscle used, no operation scar, no spider angiomata, no gynecomastia
    - Palpation: no palpable mass or nodule, no subcutaneous emphysema, no tenderness
    - Ausculation: clear breathing sound
  - Heart:
    - Percussion: normal heart size
    - Ausculation: regular heart beat, normal S1, S2, no gallop, no murmur

-
  - Abdomen:
    - Inspection: in
      - flat contour, symetrically, no scar, no stria, no dilated veins, no rashes
    - Ausculation: normoactive bowel sound, no abdominal bruits
    - palpation: soft, no muscle guarding or rigidity, no local tenderness, no rebounding pain, no palpable mass, no palpable pulsation
    - Percussion: no shifting dulllness, no hepatomegaly, no splenomagaly
  -

Peripheral pulsation :	CA	BA	RA	FA	PA	PTA	DPA
Right side :	++	++	++	++	++	++	++/+++
Left side :	++	++	++	++	++	++	++/+++

  - Genito-anus: no tenderness or palpable mass lesion or rectal shelf, normal external genitalia, normal anal tone, brown stool

CRS CONDITION

-
  - Lung

手術日期及方法

住院治療經過

- After admission, pre op survey was OK and operation was done smoothly. She tolerated the procedure well and could stand oral feeding gradually. Under relatively stable conditions, she will be discharged.

合併症

一般檢查

- 累積報告 :

DATE	TIME	ALB	TP	BIL.T	BIL.D	ALKP	AST	ALT	LDH	AMY	LIPASE	NH3	Ferritin
1100605	1342	3.9		0.83	0.21	78	23	20					
1100607	1044	3.4					19	12					

DATE	TIME	WBC	RBC	HGB	HCT	MCV	PLT	BLST	NEUT	BAND	SEG	LYM	MON	EOS	BAS
1100605	1341	13250	4.43	13.2	39.4	88.9	219		94.5			4.5	0.8	0.0	0.2
1100607	1044	8730	4.03	12.2	35.4	87.8	207		76.0			17.6	4.8	1.3	0.3

DATE	TIME	NA	K	CL	BUN	CREAT	eGFR	CA	Mg	P	CRP	ESR	PCT	D-Dimer	LACTATE
1100605	1342	142	4.0	108	8	0.63	105.47	8.7							
1100607	1044					0.73					2.812	41			

DATE	TIME	GLUCOSE	HbA1C	CHOL	TG	HDL	LDL	KETONE	CK	CKMB	TRO-T	PT-P	APTT-P	NT-pro BNP
1100605	1341	125												
1100607	1044		5.3	113	63	51	53							

DATE	TIME	SP-GR	PH	PROT	GLUC	KETON	BILI	UROB	OB	NITR	LEU	RBC	WBC	EPCEL
1100607	1051	1.007	7.0	Negative	Negative	Negative	Negative	0.2	Negative	Negative	Trace	0-2	0-2	0-1

特殊檢查

病理報告

其它

出院時情況

- 3.改門診治療

出院指示

-

藥名	劑量	劑量單位	用法	頻次	數量	數量單位	現況	天數	單位
Mag. Oxide tab	2	TAB	PO	TID	84	TAB	TH	14	天
Ultracet tab	1	TAB	PO	QID	28	TAB	TH	7	天

出院計劃

主治醫師_2062F 蔣鋒帆　　/　住院醫師_2309E 古朋育

正式摘要(電子簽章：000000218842042)

W76 / W76

doctorRank:B
